# Non-Invasive Physical Plasma for Preventing Radiation Dermatitis in Breast Cancer: A First-In-Human Feasibility Study

**DOI:** 10.3390/pharmaceutics14091767

**Published:** 2022-08-24

**Authors:** Cas Stefaan Dejonckheere, Adriana Torres-Crigna, Julian Philipp Layer, Katharina Layer, Shari Wiegreffe, Gustavo Renato Sarria, Davide Scafa, David Koch, Christina Leitzen, Mümtaz Ali Köksal, Thomas Müdder, Alina Abramian, Christina Kaiser, Andree Faridi, Matthias Bernhard Stope, Alexander Mustea, Frank Anton Giordano, Leonard Christopher Schmeel

**Affiliations:** 1 Department of Radiation Oncology, University Hospital Bonn, 53127 Bonn, Germany; 2 Institute of Experimental Oncology, University Hospital Bonn, 53127 Bonn, Germany; 3 Department of Gynaecology, Division of Senology, University Hospital Bonn, 53127 Bonn, Germany; 4 Department of Gynaecology and Gynaecological Oncology, University Hospital Bonn, 53127 Bonn, Germany

**Keywords:** non-invasive physical plasma, cold atmospheric plasma, cold atmospheric pressure plasma, tissue tolerable plasma, physical plasma medicine, radiation dermatitis, breast cancer, radiation therapy, feasibility

## Abstract

Radiation dermatitis (RD) is the most common acute side effect of breast irradiation. More than a century following the therapeutic utilisation of X-rays, potent preventative and therapeutic options are still lacking. Non-invasive physical plasma (NIPP) is an emerging approach towards treatment of various dermatological disorders. In this study, we sought to determine the safety and feasibility of a NIPP device on RD. Thirty patients undergoing hypofractionated whole-breast irradiation were included. Parallel to radiation treatment, the irradiated breast was treated with NIPP with different application regimens. RD was assessed during and after NIPP/radiation, using clinician- and patient-reported outcomes. Additionally, safety and feasibility features were recorded. None of the patients was prescribed topical corticosteroids and none considered the treatment to be unpleasant. RD was less frequent and milder in comparison with standard skin care. Neither NIPP-related adverse events nor side effects were reported. This proven safety and feasibility profile of a topical NIPP device in the prevention and treatment of RD will be used as the framework for a larger intrapatient-randomised double-blind placebo-controlled trial, using objective and patient-reported outcome measures as an endpoint.

## 1. Introduction

Early breast cancer remains the most common cancer diagnosis [1]. Treatment usually includes lumpectomy followed by adjuvant whole-breast irradiation (WBI) to improve local control and survival [2]. Radiation dermatitis (RD) is the most common acute side effect of breast irradiation, arising in up to 85% of patients and being of moderate to severe degree in up to 30% [3,4,5,6]. Not only does it impact quality of life (QOL), but treatment interruptions might be necessary in severe cases, compromising disease control outcomes [4,6]. Despite continuous research efforts, effective preventive and therapeutic alternatives are lacking. Furthermore, substantial variation in the management of RD amongst practitioners persists, as compelling guidelines are yet to be published [4,7].

Non-invasive physical plasma (NIPP) is emerging as a promising novel treatment modality for various skin conditions, such as psoriasis, eczema, diabetic ulcers, and different types of dermatitis [8,9]. Physical plasma is referred to as the fourth state of matter and is characterised by free electrons [10]. Contrary to thermal plasma, NIPP is generated using a high-frequency alternating field under atmospheric pressure, and therefore, only reaches room temperatures, rendering it safe for clinical application. Dielectric barrier discharges (DBD) are a type of NIPP, generated out of ambient air, without the need of a carrier gas [11]. The reactive mix of electrons, ions, excited atoms, reactive oxygen and nitrogen species (RONS), UV radiation, and heat that arises has been shown to positively affect tissue healing in a dose-dependent manner [12]. NIPP treatment is well-tolerated, and no side effects have been reported in previous series for other treatment purposes [13,14,15,16].

DBD-generated NIPP for preventing or treating RD has not yet been studied in a clinical setting. However, limited preclinical data in mice suggest effectiveness (i.e., delayed onset and reduced severity of RD) [17]. In the current first-in-human feasibility study, we evaluate safety and toxicity, practicality, gross costs, and preliminary efficacy. The herein obtained results will be considered as baseline for a future prospective clinical trial to evaluate the effect on incidence and severity of RD in patients undergoing WBI (DRKS00026225).

## 2. Materials and Methods

### 2.1. Participants

From October 2021 through April 2022, we conducted a monocentric, single-arm phase I study enrolling patients that were scheduled for WBI of any side. Inclusion criteria were: age > 18 years, breast-conserving surgery for breast cancer, and a fractionation regimen of 40.05 Gy in 15 fractions of 2.67 Gy with or without a sequential normofractionated boost to the tumour bed of 16 Gy in 8 fractions of 2 Gy. The exclusion criteria were defined as synchronous metastatic disease, mastectomy, breast implant reconstruction, alternative fractionation regimens, history of ipsilateral breast irradiation, any pre-existing dermatological disorders, active dermatitis, current treatment with topical or oral corticosteroids, and patient refusal to participate. A written informed consent was obtained from all included participants. This study was conducted in accordance with the Declaration of Helsinki, and the protocol was approved by the local Institutional Review Board on 29 September 2021 (210/21).

### 2.2. Radiation Protocol

All treatment plans were generated with 6-MV or combined energies up to 10-MV photons, either with tangential intensity-modulated radiotherapy (IMRT) beams or volumetric modulated arc therapy (VMAT). In invasive cancer cases, a sequential normofractionated boost to the tumour bed (16 Gy in 8 fractions of 2 Gy) was applied in patients with positive margins following surgical resection, patients aged 50 and younger, and patients aged above 51 in case of a high grade tumour (≥ pT2, HER2/neu positive, triple-negative, G3).

Contouring was carried out following the ESTRO consensus guideline [18]. Treatment planning aimed to achieve a homogenous dose distribution in the target volume. The International Commission on Radiation Units and Measurements (ICRU) recommendations for dose limits of 95% to 107% were applied.

All patients were treated on a TrueBeam STx (Varian Medical Systems, Palo Alto, CA, USA) linear accelerator in a supine position with both arms up. Left-sided WBI was performed in deep inspiration breath-hold (DIBH) for compliant patients.

### 2.3. Standard Skin Care

Standard skin care with a urea-based lotion (Eucerin UreaRepair PLUS 5% Urea Lotion, Beiersdorf, Hamburg, Germany) was applied to the whole breast. All patients received oral and written information to apply the urea lotion twice daily, from the first day of treatment onwards, according to institutional standards. In addition, they were encouraged not to use any complementary topical treatment. Compliance was checked during the scheduled patient visits. Patients presenting with grade ≥ 2 RD with moist desquamation and severe pain were prescribed topical corticosteroids if required until symptoms resolved.

### 2.4. NIPP Protocol

**Figure 1:**
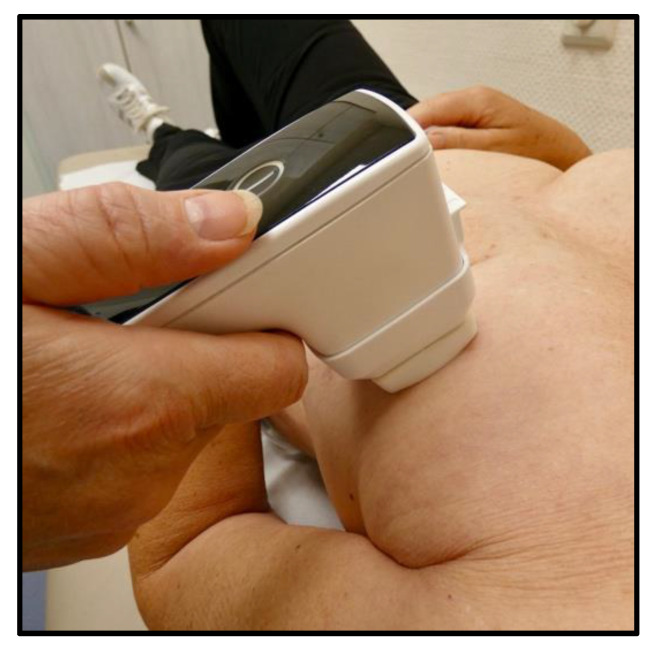
Generation and application of NIPP to the breast. The device is pressed loosely on the skin with a separate 4 × 4 cm spacer for each patient and each visit. NIPP = non-invasive physical plasma.

To generate and apply the NIPP, a wireless topical plasma generator (plasma derma care, terraplasma medical, Garching, Germany) was employed. The device was pressed loosely on the skin with a 4 × 4 cm spacer per patient and visit, to achieve an optimal and reproducible distance to the skin (Figure 1). This process was repeated in all patients until the entire breast surface was treated. Three different treatment durations and frequencies were explored: 60 s of NIPP administration twice a week, 180 s three times a week, and 120 s five times a week, using the device’s pre-set program ensuring a constant dose of NIPP (defined as a constant dose of ozone, used as a surrogate for RONS) [19].

### 2.5. Patient Evaluation

Patients were evaluated at the routine weekly on-treatment visits. Any additional visits requested by the participants were also recorded. RD was assessed according to the National Cancer Institute’s Common Terminology Criteria for Adverse Events (CTCAE) v5.0: grade 0 = no radiation dermatitis; grade 1 = faint erythema or dry desquamation; grade 2 = moderate to brisk erythema, patchy moist desquamation, mostly confined to skin folds and creases, moderate oedema; grade 3 = moist desquamation in areas other than skin folds and creases, bleeding induced by minor trauma or abrasion; grade 4 = life-threatening consequences, skin necrosis or ulceration of full thickness dermis, spontaneous bleeding from involved site, skin graft indicated; grade 5 = death [20]. Upon completion of radiotherapy, toxicity was graded by an experienced breast radiation oncologist.

By the end of treatment, as well as during the first follow-up visits two and six weeks after treatment completion, the patient-assessed modified Radiation-Induced Skin Reaction Assessment Scale (RISRAS) was recorded [21]. All patients reported their maximum breast-related experience of pain, itching, burning, as well as limitations in daily activities. All items were scored on a 4-point Likert scale: 0 = not at all; 1 = a little; 2 = quite a bit; 3 = very much. Furthermore, the subjective experience of each patient was monitored with four yes-no statements (Table 1).

### 2.6. Feasibility

Besides clinical outcomes, safety and potential harm, practicality (from both patients’ and physicians’ point of view), as well as gross costs were documented, in order to assess the overall feasibility of NIPP in preventing and/or treating RD in breast cancer patients undergoing WBI. Operator satisfaction was defined as the user’s comfort with and acceptability of the device studied. Adverse device events (ADEs) were defined as unexpected adverse events related to the use of the investigational medical device, including those resulting from insufficiencies or inadequacies in the instructions for use, the deployment, the operation, or any malfunction.

## 3. Results

### 3.1. Patient Characteristics

**Figure 2:**
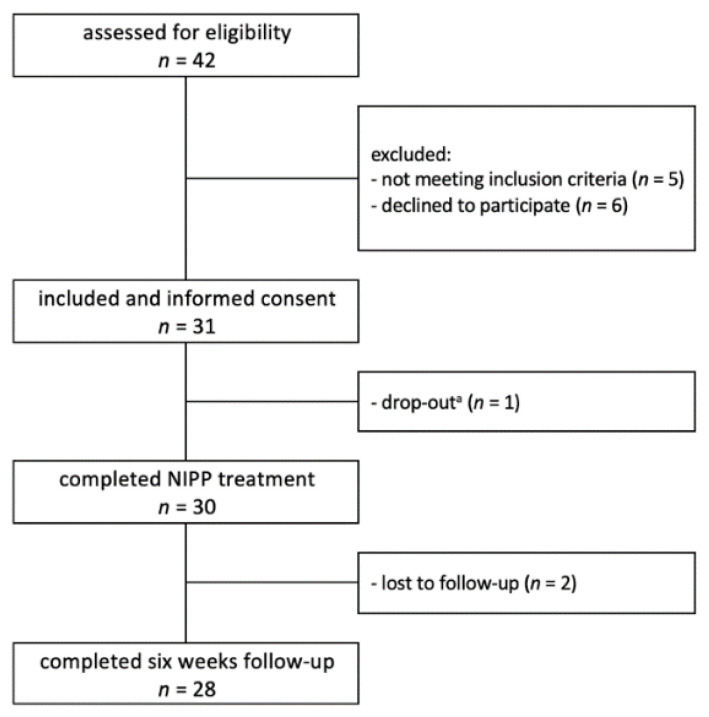
Flowchart of patient selection. NIPP = non-invasive physical plasma. ^a^ Interruption of radiation treatment for 7 days due to community-acquired pneumonia.

A flowchart of patient selection and inclusion is shown in Figure 2. Patient and treatment characteristics are summarised in Table 2. A total of 30 patients were included (90.0% Caucasian; 96.7% female), with a median age of 56 (30–83) years. Fitzpatrick II was the most common skin type, present in 76.7% of patients. Neoadjuvant or concomitant chemo- and/or immunotherapy were administered in 26.7%, and hormone therapy was prescribed in 83.3%. Half of all patients had an indication for a sequential boost to the tumour bed. The median breast planning target volume (PTV) was 798 (129–1771) mL, whereas the median boost PTV was 126 (52–307) mL.

### 3.2. NIPP Application Regimens

To determine the optimal treatment duration and frequency, different application regimens were explored. In the first 3 patients (10%), treatment time was 60 s twice a week. No dose-limiting toxicities (DLTs) were observed, i.e., no increase in RD severity compared to standard skin care up to six weeks of follow-up. Because of the excellent tolerance in these patients, treatment time was increased to 180 s three times a week (80%). In the last 3 patients (10%), a further dose intensification to 120 s five times a week was investigated.

### 3.3. Severe Adverse Reactions

No severe adverse reactions or interactions deemed related to NIPP therapy, but not radiation therapy, were observed, regardless of the duration or frequency of NIPP applications.

### 3.4. Acute Skin Toxicity as Assessed by Clinicians (Clinician-Reported Outcome)

**Figure 3:**
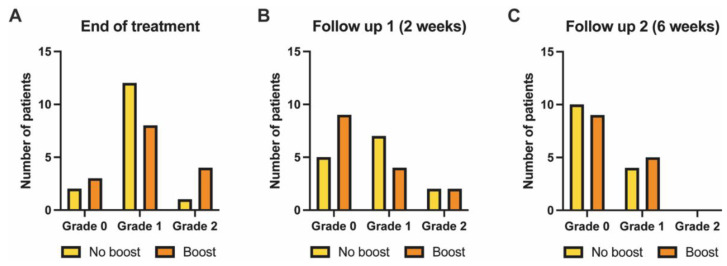
Maximum severity of radiation dermatitis (**A**) upon NIPP and radiation treatment completion and (**B**) at two and (**C**) six weeks follow-up, stratified by absence (yellow) or presence (orange) of a sequential normofractionated boost. Grading according to the National Cancer Institute’s Common Terminology Criteria for Adverse Events (CTCAE) v5.0 [20]. NIPP = non-invasive physical plasma.

At the time of treatment completion, 13.3% of patients in the no-boost group (*n* = 15) had no RD, while 80% and 6.7% developed grade 1 and grade 2 RD, respectively. In the boost group, these rates were 20%, 53.3%, and 26.7%. No grade ≥ 3 RD was observed (Figure 3A). Dry desquamation was seen in only 2 patients (6.7%) and moist desquamation in 1 patient (3.3%), all occurring in the boost group.

Two weeks after treatment completion, the rate and severity of RD was grade 0 in 35.7%, grade 1 in 50%, and grade 2 in 14.3% within the no-boost group; proportionally, these rates were 60%, 26.7%, and 13.3% in the boost group (Figure 3B).

At six weeks following breast irradiation, grade 2 RD was not observed anymore. The incidence of RD in the no-boost group was 71.4% and 28.6% for grade 0 and grade 1 RD, respectively. In the boost group, these incidences were 64.3% and 35.7% (Figure 3C).

None of the patients was prescribed topical corticosteroids during radiotherapy or follow-up period.

**Figure 4:**
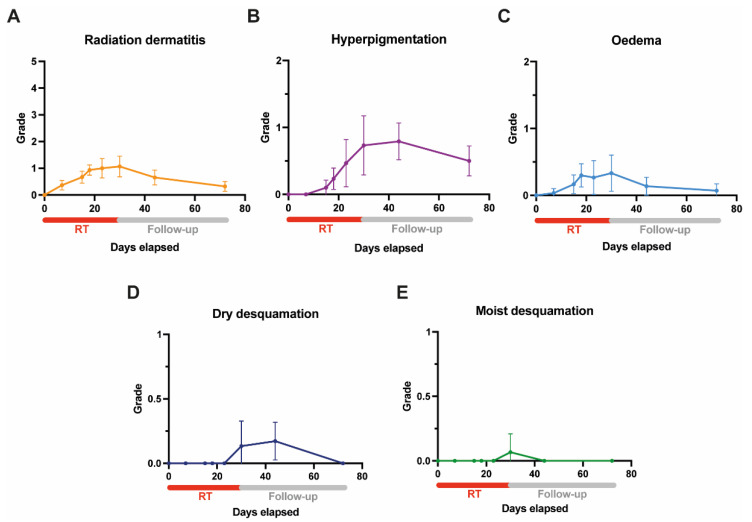
Evolution of mean acute toxicity for the entire cohort during (routine weekly on-treatment visits) and after (two- and six-weeks follow-up) adjuvant whole-breast irradiation in combination with NIPP: (**A**) radiation dermatitis, (**B**) hyperpigmentation, (**C**) oedema, and (**D**) dry and (**E**) moist desquamation. Grading according to the National Cancer Institute’s Common Terminology Criteria for Adverse Events (CTCAE) v5.0 [20]. Error bars indicate 95% confidence intervals. NIPP = non-invasive physical plasma; RT = radiation treatment.

Figure 4A–E shows the evolution of acute toxicity (RD, hyperpigmentation, oedema, and dry and moist desquamation) during and after adjuvant radiation treatment in combination with NIPP.

### 3.5. Acute Skin Toxicity and Treatment Experience as Assessed by Patients (Patient-Reported Outcome)

The results of the patient-assessed modified RISRAS questionnaire, upon treatment completion, are shown in Table 3.

Regarding the subjective patient experience of the NIPP treatment, none of the patients found the treatment to be unpleasant, whereas all would recommend NIPP treatment to a friend undergoing irradiation for breast cancer. Outside of this study, 93.1% would also have wanted to be treated with NIPP during WBI (3.4% are undecided) and 27.6% believed that their symptoms had been reduced by the NIPP treatment (51.7% do not know).

### 3.6. Handling and Practicality

There were no ADEs (i.e., no insufficiencies or inadequacies in the instructions for use, deployment, or operation), and no malfunctions were reported. Operator satisfaction was high as the investigational NIPP device is easy to use and can be rapidly charged in between patients using the included docking station.

### 3.7. Cost Estimation

NIPP can be applied by a single healthcare professional or prospectively even by patients themselves and requires only minimal training as the device is user-friendly and self-explanatory.

The plasma derma care device set is offered for EUR 2900.00. A single 4 × 4 cm spacer, which is needed for NIPP application, costs EUR 14.00 and can be used up to 10 min. A detailed overview of the estimated weekly number of spacers, spacer cost, and additional treatment time, for the different treatment durations and frequencies, is provided in Table 4.

## 4. Discussion

Even though RD is the most common acute side effect of breast irradiation and in radiation oncology overall, there has been little progress in the development of new topical preventative and treatment agents. This results in a significant physical and psychological impact among a large proportion of patients affected by RD. While topical corticosteroids are effective in reducing RD-induced symptoms, widespread use remains limited due to its side effect profile [4]. Despite some experimental products showing promising results in RD prevention or management, barely any of them proved efficacious in randomised trials [22]. Reasons for this predicament are manifold, but primarily, there are two sets of factors. Firstly, studies on RD often encompass a heterogeneous patient population with either varying dose-fractionation regimens or treatment sites, or lack comparison groups, not to mention placebo controls. A second issue is the subjectivity of physician-assessed grading of RD such as CTCAE with high inter- and intra-observer variability. This may also be accompanied by significant discrepancies with patient-reported outcomes, e.g., when evaluating the impact of RD on QOL [23,24,25]. There is a need for objective RD assessment methods, while simultaneously acknowledging the patient’s perspective, in order to investigate future prevention and treatment strategies.

As NIPP is emerging as a promising novel treatment modality for a number of skin conditions and positively affects tissue healing without side effects, it is of major interest to test its clinical application and value in RD. In this first-in-human feasibility study, we therefore investigated the safety, practicality, and gross costs of a topical NIPP-based prevention and treatment method for RD in breast cancer irradiation.

RONS play a crucial role in tissue damage induced by ionising radiation [26]. However, in the context of NIPP therapy, RONS are also significantly involved in tissue healing and regeneration, necessitating a feasibility and dose escalation study for the combination of NIPP and radiation [12]. In vitro and in vivo studies have shown that NIPP application to human skin cells does not result in any impairment of cell physiology, cytology, nor DNA integrity, making it safe for clinical application [13,15,27,28,29]. Overall, the use of NIPP in the current study was safe and practical from both the patient’s and the physician’s point of view, along with excellent patient compliance.

A preclinical placebo-controlled trial in irradiated mice showed delayed onset and reduced severity of RD [17]. These promising initial observations were confirmed in the current study, at least for the time being: grade ≥ 2 RD, dry and moist desquamations were less frequent in comparison with recent high-quality toxicity assessments in contemporary hypofractionated WBI. The patient-reported outcomes also indicate a reduced incidence of pain, itching, and burning following NIPP treatment compared to standard skin care. Table 5 compares clinician- and patient-reported outcomes of the current study and selected previous studies on acute toxicity following hypofractionated WBI [3,6,30]. The clinical benefit of these differences, i.e., an actual effect of NIPP application, however, cannot be accurately determined yet. This relates to the relatively small sample size and the use of NIPP as a potential add-on treatment. Additionally, the use of visual and thus subjective physician-assessed RD grading such as CTCAE does not suffice to establish its exact role [31,32,33].

The individual spacer size is relatively small, resulting in a considerable cumulative treatment time, especially for women with larger breast size. Because of the excellent initial tolerance with 60 s twice a week, the treatment time and frequency were increased to 180 s three times a week and subsequently 120 s five times a week (i.e., daily), in order to identify the optimal dose and interval. We hypothesise that an additional dose escalation step might yield an even better outcome, since it has been shown that longer treatment times generate more ozone, used as a surrogate for RONS, key players in bacterial decolonisation, which might prevent acute RD [19,34]. Furthermore, with increased NIPP treatment duration, the secretion of anti-inflammatory and regenerative signalling molecules is also promoted, which could also lead to a better outcome [16]. The current device, however, is rather unsuitable to accommodate for this, since the spacer size is a limiting factor. A spacer with a larger treatment area, encompassing larger areas of the breast would deem useful and could reduce the total treatment time while delivering the same dose of NIPP, resulting in a similar outcome. The device was, however, designed to treat smaller surfaces such as chronic wounds, which are more likely to fit within the dimensions of a single spacer. Furthermore, the use of multiple spacers for a single treatment session yields an additional cost. If fewer or a single spacer could be used for individual treatment sessions, it would significantly reduce the cost, and also entail a more sustainable treatment method.

The pathophysiology of RD is complex and the signalling pathways and mechanisms in which NIPP positively affects tissue healing are not yet fully understood. One possible explanation might lie in bacterial decolonisation. Ionising radiation disrupts skin barrier function through direct DNA damage in epidermal basal layer cells or indirect DNA damage due to secondary formation of RONS [35,36]. Microorganisms or microbial antigens may subsequently trigger an inflammatory response, which enhances the clinical appearance of RD through an increased immune reaction. This also impairs the repair of the epidermal barrier and further prolongs RD. A recent report underpins this hypothesis: the local use of chlorhexidine body wash once daily significantly lowered median RD severity and prevented moist desquamation in patients undergoing breast irradiation when compared to standard skin care [34]. NIPP may work in a similar fashion by reducing the bacterial load on the irradiated skin, thereby mitigating the local inflammatory reaction, since the highly effective antimicrobial activity of NIPP is well-documented [37,38].

A further mechanism might be that NIPP promotes the proliferation and migration of keratinocytes, fibroblasts, and endothelial cells, thus facilitating tissue recovery [39]. In the latter, tube formation is accelerated, improving vascular shear stress and contributing to angiogenesis. These new vascular networks lead to enhanced capillary blood flow and result in increased local oxygen saturation and nutrient supply [40,41]. Furthermore, NIPP induces the expression of genes relevant to proper wound healing in immune cells (e.g., type I collagen, transforming growth factor-beta, and alpha-smooth muscle actin) [42].

An additional possible effect of NIPP on tissue healing includes significant changes to the human skin barrier lipid stoichiometry [43]. The relevance of this in the light of skin barrier regulation, however, is still poorly understood.

An analysis of the potential differences in efficacy between the different NIPP treatment duration and frequency subgroups is not possible at this time due to the small sample size. The main scope of the current study was safety, tolerability, and feasibility; therefore, a thorough assessment of the (possibly dose-dependent) efficacy will be performed in a randomised, prospective fashion. Patient recruitment is ongoing to elucidate the actual clinical value of NIPP in WBI-associated RD management. Additionally, the assessment of biochemical markers of inflammation could be of added value.

## 5. Conclusions and Future Perspectives

DBD-generated NIPP proved to be safe and feasible with no DLT events in this cohort of patients undergoing adjuvant WBI. An ongoing randomised double-blind placebo-controlled trial to evaluate the effect of NIPP on RD will elucidate the clinical value of this approach in a larger number of patients.

## Figures and Tables

**Table 1:** The subjective experience of patients was monitored with four yes-no statements.

1. I found the NIPP treatment to be unpleasant.
2. I would recommend NIPP treatment to a friend undergoing irradiation for breast cancer.
3. I would also have wanted to be treated with NIPP to potentially prevent and/or treat RD outside of this study.
4. I have the impression that my symptoms have been reduced by the NIPP treatment.

NIPP = non-invasive physical plasma; RD = radiation dermatitis.

**Table 2:** Summary of patient and treatment characteristics (*n* = 30).

Median Age (Range) in Years	56 (30–83)
Sex	*n* (%)
female	29 (96.7)
male	1 (3.3)
Ethnicity	*n* (%)
Caucasian	27 (90.0)
other	3 (10.0)
Fitzpatrick skin type	*n* (%)
I	4 (13.3)
II	23 (76.7)
III	3 (10.0)
Side	*n* (%)
left	16 (53.3)
right	14 (46.7)
pT-stage	*n* (%)
Tis	6 (20)
T1	19 (63.3)
T2	4 (13.3)
T3	1 (3.3)
pN-stage	*n* (%)
N0	25 (83.3)
N1	5 (16.7)
Boost	15 (50.0)
Median PTV Breast (mL)	798 (129–1771)
Median PTV Boost (mL)	126 (52–307)

pT = pathological stage of the primary tumour; Tis = carcinoma in situ; pN = pathological stage of the regional lymph nodes; PTV = planning target volume.

**Table 3:** Results of the patient-assessed modified Radiation-Induced Skin Reaction Assessment Scale (RISRAS) questionnaire upon treatment completion [21]. All items were scored on a 4-point Likert scale.

*n* (%)	Not at All	a Little	Quite a Bit	Very Much
**Pain**	16 (53.3)	12 (40)	2 (6.6)	0 (0)
**Itching**	19 (63.3)	10 (33.3)	1 (3.3)	0 (0)
**Burning**	25 (83.3)	5 (16.7)	0 (0)	0 (0)
**Limitations in ADL**	23 (76.7)	5 (16.7)	2 (6.6)	0 (0)

ADL = activities of daily living.

**Table 4:** Estimated weekly number of spacers, spacer cost, and additional treatment time (excluding possible waiting time and time needed to replace used spacers) for a mean breast surface area of 12 × 16 cm (i.e., 3 × 4 spacers). The grey areas indicate the three different treatment duration and frequency combinations that were explored in the current study. Cost and time estimates may vary depending on breast size.

			NIPP Treatment Frequency Per Week				
			1	2	3	4	5
**NIPP treatment duration ^a^**	**60 s**	spacers/w	1–2	2–4	3–6	4–8	5–10
		cost/w	EUR 14–28	EUR 28–56	EUR 42–84	EUR 56–112	EUR 70–140
		time/w	10–20 min	20–40 min	30–60 min	40–80 min	50–100 min
	**120 s**	spacers/w	2–3	4–6	6–9	8–12	10–15
		cost/w	EUR 28–42	EUR 56–84	EUR 84–126	EUR 112–168	EUR 140–210
		time/w	20–30 min	40–60 min	1–1.5 h	80–120 min	100–150 min
	**180 s**	spacers/w	3–4	6–8	9–12	12–16	15–20
		cost/w	EUR 42–56	EUR 84–112	EUR 126–168	EUR 168–224	EUR 210–280
		time/w	30–40 min	60–80 min	1.5–2 h	120–160 min	150–200 min

^a^ Per 4 × 4 cm area of breast skin. NIPP = non-invasive physical plasma; w = week.

**Table 5:** Comparison between the current study and selected previous studies of acute toxicity incidence and severity upon treatment completion following hypofractionated whole-breast irradiation, including assessment of PRO.

		RD Grade ^b^			Desquamation		PRO			
**Author (year)**	*n*	0	1	≥2	dry	moist	pain	itching	burning	ADL limitations
Current study (2022)	30	17%	67%	17%	7%	3%	0.53 ± 0.63 ^c^ (47%)	0.40 ± 0.56 ^c^ (36%)	0.17 ± 0.38 ^c^ (17%)	0.30 ± 0.60 ^c^ (23%)
Schmeel et al. (2020) [6]	70 ^a^	21%	51%	27%	34%	9%	0.62 ± 0.66 ^c^	0.89 ± 0.93 ^c^	0.63 ± 0.68 ^c^	0.24 ± 0.66 ^c^
Shaitelman et al. (2015) [3]	138 ^a^	6%	58%	36%	N/A	N/A	55%	54%	N/A	N/A
Jagsi et al. (2015) [30]	578 ^a^	6%	67%	27%	19%	7%	72%	37%	16%	7%

^a^ Number of patients in the hypofractionation arm. ^b^ CTCAE v5.0 for the current study and CTCAE v4.0 for the others. ^c^ Mean modified RISRAS ± standard deviation. RD = radiation dermatitis; PRO = patient-reported outcome; ADL = activities of daily living; N/A = not available.

## Data Availability

Not applicable.
